# EEG Subject Identification and Open-Set Rejection Across Paradigms

**DOI:** 10.3390/s26175471

**Published:** 2026-08-29

**Authors:** Cai Chen, Danyang Lv, Jiazheng Sun, Lijun Liu, Shuxian Li, Mufeng Pan, Chongxuan Tian, Zhi Li, Fengxia Wu, Ningling Zhang, Tao Jing

**Affiliations:** 1 Brain Computer Interface Research Center, Shandong Institute of Advanced Technology, Chinese Academy of Sciences, Jinan 250000, China; d202582315@hust.edu.cn (C.C.); dy.lv@sdiat.ac.cn (D.L.); jz.sun@sdiat.ac.cn (J.S.); lj.liu@sdiat.ac.cn (L.L.); 2 State Key Laboratory of Environment Health (Incubation), Key Laboratory of Environment and Health, Ministry of Education, Key Laboratory of Environment and Health (Wuhan), Ministry of Environmental Protection, School of Public Health, Tongji Medical College, Huazhong University of Science and Technology, #13 Hangkong Road, Wuhan 430030, China; m202575905@hust.edu.cn (S.L.); m202575925@hust.edu.cn (M.P.); 3 School of Control Science and Engineering, Shandong University, Jinan 250000, China; 202420777@mail.sdu.edu.cn (C.T.); 201814514@mail.sdu.edu.cn (Z.L.); 4 School of Basic Medical Sciences, Shandong University, Jinan 250012, China

**Keywords:** electroencephalography, biometric identification, multi-paradigm, open-set rejection, performance evaluation

## Abstract

**Objective**: To compare electroencephalography (EEG)-based subject-identification and open-set rejection performance across experimental paradigms while examining model robustness and cross-session generalization. **Methods**: The M3CV database was analyzed across resting state, transient sensory stimulation, steady-state sensory stimulation, P300 Oddball, and motor execution. Closed-set identification was evaluated across feature representations, conventional machine-learning models, and raw-EEG deep-learning baselines. Open-set performance was further assessed using an identity-first leakage-free nested procedure with 60 enrolled identities, 15 development unknown identities, and 20 final-test unknown identities. **Results**: Under the P4 within-session protocol, transient sensory stimulation achieved the highest Rank-1 accuracy (98.10%). Differential entropy and shrinkage linear discriminant analysis achieved mean Rank-1 accuracies of 98.01% and 98.29%, respectively. Under leakage-free nested open-set evaluation, transient sensory stimulation achieved DIR@FPIR = 5%, DIR@FPIR = 1%, and AU-OSCR values of 91.75%, 77.32%, and 97.36%, respectively. Deep-learning analyses confirmed strong within-session identity discrimination but showed model-dependent open-set paradigm rankings. In contrast, strict cross-session transfer produced marked degradation across all model families, with Rank-1 accuracy falling to approximately 1.9–5.4% and verification AUC to approximately 0.53–0.56; unsupervised target normalization provided little recovery. **Conclusions**: EEG paradigms exhibited strong but largely session-dependent identity discriminability. Transient sensory stimulation provided the most favorable within-session open-set performance under the primary conventional framework, whereas cross-session variability dominated paradigm-dependent differences under zero-shot transfer.

## 1. Introduction

With the expansion of digital services, telemedicine, and human–computer interaction systems, identity recognition has become increasingly important for information security and privacy protection. Conventional biometric traits, including fingerprints, facial characteristics, iris patterns, and voice, are widely used but may remain vulnerable to trait exposure, replication attacks, or non-consensual acquisition. Electroencephalography (EEG) originates from living neural activity and offers relative concealment, resistance to forgery, renewability, and potential liveness-related information; it is, therefore, regarded as a promising emerging biometric modality. Recent EEG-biometric research has progressed from feasibility demonstrations toward feature stability, temporal generalization, secure authentication, and practical evaluation [1]. Nevertheless, long-term stability, user adaptability, and robust decision boundaries remain unresolved, and stimulation conditions, signal representations, and model architectures may all alter the expression of identity-related EEG information [2].

EEG-based identification performance depends not only on the algorithm but also on the experimental paradigm. Different paradigms engage distinct neural processes and may generate identity information with different strengths and levels of stability. Resting-state paradigms are simple and impose little user burden, but they may be affected by spontaneous state fluctuations. Visual and auditory sensory paradigms can strengthen externally driven responses; P300 and other event-related potentials reflect individual differences in stimulus evaluation and attentional processing; and motor-related paradigms may contain subject-specific patterns associated with movement execution or intention. Recent studies have improved visual-evoked-potential biometrics by combining multiple visual stimuli [3], enhanced VEP feature robustness using a dual-attention refinement network [4], and compared EEG authentication across multiple event-related-potential conditions [5]. In addition, a public EEG biometric dataset containing resting-state and auditory-evoked conditions has provided a basis for evaluating multiple stimulus paradigms [6]. However, most previous work has focused on one paradigm or a limited set of stimuli, and systematic comparisons under a common data source, sample budget, and evaluation protocol remain scarce.

EEG biometric representations have also evolved from conventional frequency-domain statistics to spatial–frequency modeling, functional-connectivity representations, and deep feature learning. Tensorized spatial–frequency attention combined with domain adaptation has been applied to cross-session EEG biometrics [7]; functional-connectivity graphs have been evaluated using graph neural networks [8]; and EEG topographic maps have been incorporated into multi-view fusion models [9]. Although these methods have advanced algorithmic performance, comparisons among paradigms can be confounded by feature dimensionality, model complexity, channel configuration, and sample partitioning. If paradigms are evaluated using unequal sample sizes, different train–test partitions, or randomly mixed temporally adjacent epochs, the observed performance differences may not accurately reflect the identity-discriminative information inherent to the paradigms themselves.

Most EEG-biometric studies have used closed-set identification or known-user verification, assuming that every test sample belongs to an enrolled identity. Such evaluations quantify discrimination among known identities but do not reproduce the practical situation in which an unregistered person enters the system. A realistic identification system must not only recognize legitimate enrolled users but also reject unknown inputs rather than forcing them into one of the enrolled classes. Recent studies have, therefore, examined cancelable multimodal EEG authentication [10], fuzzy-logic-based biometric identification [11], reusable EEG recognition with fuzzy extractors [12], and datasets designed to evaluate the effects of authentication protocols [13]. These developments suggest that closed-set accuracy or equal error rate alone is insufficient for determining whether an EEG paradigm is suitable for practical authentication; open-set rejection should be treated as an essential component of evaluation.

Experimental-task design and acquisition burden also influence the practical utility of EEG biometrics. Multitask EEG identification using an autoencoder–CNN framework has demonstrated that task conditions can alter the identity-discriminative information learned by a model [14]. The central question has, therefore, shifted from whether EEG can distinguish individuals to which paradigms can provide stable, secure, and practically useful identity information under fair evaluation. Multi-paradigm studies must control sample-size differences, avoid potential leakage caused by randomly splitting adjacent temporal windows, and evaluate both closed-set identification and open-set rejection. Recent advances in EEG biometric research have also emphasized the importance of evaluation robustness beyond within-session identification. In particular, deep-learning-based representation learning, connectivity-based brainprint analysis, and cross-session adaptation approaches have expanded the capability of EEG biometric systems [15]. However, these approaches are often evaluated under different datasets, paradigms, preprocessing pipelines, or partition strategies, making direct comparison among experimental paradigms difficult.

To address these issues, this study systematically compared resting state, transient sensory stimulation, steady-state sensory stimulation, P300 Oddball, and motor execution using a unified multi-paradigm EEG dataset. Subject- and paradigm-level sample balancing was combined with non-overlapping temporally ordered training and test blocks to reduce optimistic bias arising from random partitioning of adjacent EEG segments. Closed-set performance was evaluated across feature representations, machine-learning models, and temporal enrollment protocols, and the contributions of individual tasks within each paradigm were examined. Open-set identification and unknown-identity rejection were then assessed by strictly separating enrolled identities, development unknown identities, and final-test unknown identities.

Furthermore, deep-learning baselines and cross-session generalization analyses were incorporated to evaluate whether learned EEG representations could maintain identity discrimination beyond conventional feature-based approaches and recording sessions. The study aimed to provide evidence for experimental-paradigm selection and evaluation-protocol design in EEG biometrics.

## 2. Related Work

Early EEG biometric studies mainly relied on handcrafted signal representations, including spectral characteristics, entropy measures, and statistical descriptors, combined with conventional machine-learning classifiers. These approaches demonstrated the feasibility of identifying individual differences from EEG recordings and provided interpretable biometric representations. However, many studies focused on a single experimental paradigm or closed-set identification scenarios, which limited the understanding of how task conditions influence biometric stability. With the development of deep neural networks, EEG biometric systems have increasingly adopted convolutional architectures, attention mechanisms, and representation-learning strategies. Deep models can automatically capture temporal and spatial EEG patterns without manually designed features. Previous studies have explored domain adaptation, attention-based networks, and multi-view fusion strategies for EEG identification [7,9]. Nevertheless, deep-learning performance is often affected by dataset scale, session variability, and evaluation protocol. Comparisons between deep-learning representations and conventional feature-based methods under identical biometric protocols remain limited. Beyond conventional EEG feature representations, brainprint studies have introduced individual identification methods based on functional connectivity patterns and network-level neural characteristics. Connectivity-based representations provide a different perspective by characterizing stable individual neural organization rather than isolated signal features. Although brainprint approaches have demonstrated promising identification capability, their evaluation has generally focused on specific datasets or paradigms. Systematic comparisons between brainprint-inspired representations, conventional EEG features, and deep-learning approaches remain insufficient.

For practical biometric deployment, an EEG system must not only identify enrolled users but also reject unknown identities and remain robust against recording-session variability. However, many previous studies primarily report closed-set accuracy or verification metrics, where unknown identities are not explicitly considered. In addition, session-dependent variations caused by electrode placement, physiological state, and recording conditions can reduce the reliability of EEG biometric systems. Therefore, leakage-controlled open-set evaluation and cross-session testing are necessary for assessing the practical applicability of EEG biometrics. Different from previous studies focusing on individual algorithms or specific EEG paradigms, this work establishes a unified evaluation framework across five EEG experimental paradigms using the M3CV multi-subject and multi-session dataset. The proposed framework integrates balanced sampling, leakage-controlled feature and classifier evaluation, closed-set identification, open-set unknown identity rejection, deep-learning benchmarking, and cross-session analysis. This design enables a systematic investigation of how experimental paradigms affect EEG biometric performance under controlled and practically relevant conditions.

## 3. Materials and Methods

### 3.1. Study Overview

**Figure 1:**
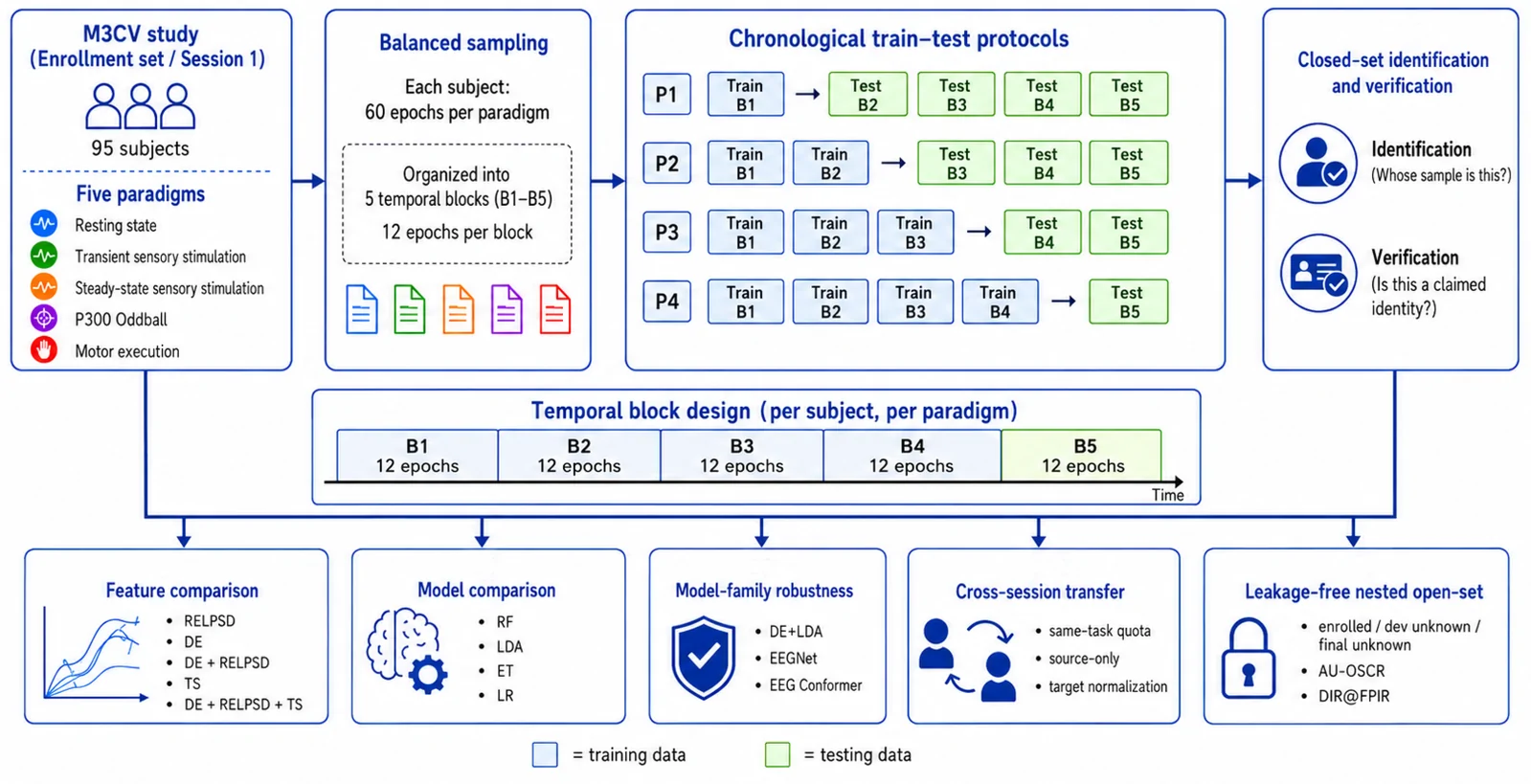
Overall study workflow and temporal-block partitioning. Each subject contributed 60 epochs per paradigm, which were organized into five non-overlapping temporal blocks (B1–B5). Closed-set and open-set analyses used temporally separated data according to the predefined protocols.

The study evaluated differences among EEG experimental paradigms in closed-set subject identification and open-set unknown-identity rejection. As illustrated in Figure 1, the workflow comprised data screening and paradigm definition, balanced sampling and temporal-block construction, closed-set identification, feature and model comparisons, within-paradigm task analysis, and open-set identification and rejection. Rather than randomly mixing epochs from the same recording session, non-overlapping blocks were constructed chronologically within each subject and paradigm. Earlier blocks were used for training and later blocks for testing. EEG-biometric research has increasingly emphasized cross-task variation, temporal changes, and protocol design [7,13]; accordingly, temporal separation was treated as a central control for fair paradigm comparison.

The closed-set analyses comprised comparisons of five paradigms under four temporally ordered training protocols, comparisons among feature representations and conventional machine-learning models, within-session auxiliary verification, statistical testing, robustness ranking across predefined analytical pipelines, and within-paradigm task-contribution analysis. The open-set analyses further separated enrolled identities, development unknown identities, and final-test unknown identities into mutually exclusive groups. To assess the robustness and generalizability of the paradigm comparison, three additional analyses were incorporated. First, EEGNet and EEG Conformer were introduced as deep-learning baselines under matched within-session closed- and open-set protocols. Second, a leakage-free nested procedure was used to perform feature and classifier selection exclusively within the enrolled-identity subset before threshold calibration and final testing. Third, strict zero-shot Session 1-to-Session 2 transfer was evaluated using exact task-matched target-session sampling, together with same-pipeline and normalization-sensitivity controls. These analyses were designed to distinguish within-session identity discriminability, open-set separability, and cross-session invariance.

### 3.2. Data Source and Experimental-Paradigm Definition

The publicly available M3CV (Multi-Subject, Multi-Session, and Multi-Task Database for EEG-Based Biometrics Challenge) database was used. M3CV contains multiple subjects, recording sessions, and experimental tasks and was established to support EEG-based individual-difference analysis, subject identification, and verification [16]. Previous work has shown that sensory stimulation, event-related potentials, and motor-related EEG can all carry identity-discriminative information [3,5,6]; however, fair comparison requires a common data source and evaluation protocol.

The Enrollment subset (Session 1) was used for the primary within-session analyses and provided complete identity labels for 95 subjects. This design allowed paradigm-dependent identity discriminability and open-set rejection to be evaluated under temporally separated within-session conditions. In addition, the labeled portion of the Testing subset was used as an independently recorded target session for the cross-session analysis. Session 2 data were used only in the dedicated cross-session experiments described in Section 3.7 and did not alter the primary Session 1 paradigm-comparison protocol. Thus, the revised study distinguishes within-session temporal generalization from true session-to-session transfer.

According to the Enrollment metadata, Tasks 1–13 were grouped into five paradigms: resting state (Tasks 1–2), transient sensory stimulation (Tasks 3–5), steady-state sensory stimulation (Tasks 6–8), P300 Oddball (Tasks 9–10), and motor execution (Tasks 11–13). Because the numbers of tasks and available epochs differed among paradigms, a common subject-level sample budget was imposed to prevent paradigms with more available data from receiving an artificial advantage. For each subject, 60 epochs were selected from each paradigm and organized into five non-overlapping temporal blocks (B1–B5), with 12 epochs per block. Within each subject and task, epochs were first ordered according to the temporal order represented by EpochID, divided into five consecutive temporal segments, and sampled without replacement. Fixed task-specific quotas were used within each 12-epoch block: 6/6 epochs for Tasks 1/2 in resting state; 4/4/4 for Tasks 3/4/5 in transient sensory stimulation; 3/4/5 for Tasks 6/7/8 in steady-state sensory stimulation; 5/7 for Tasks 9/10 in P300 Oddball; and 4/4/4 for Tasks 11/12/13 in motor execution. The selected task-level samples were then combined to form each paradigm-level temporal block. Sampling was independently repeated 10 times. Within each repetition, the same sampling manifest was used across feature representations, classifiers, and paradigms. All 95 Enrollment subjects satisfied the 60-epoch-per-paradigm requirement.

### 3.3. EEG Processing and Feature Extraction

A uniform analytical preprocessing and feature-extraction pipeline was applied to all five paradigms. Each distributed M3CV EEG epoch contained 64 channels sampled at 250 Hz and 1000 temporal samples, corresponding to a 4-s segment. The temporal mean was removed independently from each channel before feature extraction to reduce DC-offset effects. Except for the explicitly designated target-normalization sensitivity analysis described in Section 3.7, test data were not used for feature selection, supervised parameter estimation, model fitting, or threshold selection. Spatial–frequency representations have previously shown substantial value for EEG-based biometric recognition [7]. Therefore, differential entropy, relative power spectral density, and time-statistical features were evaluated under the same protocol.

#### 3.3.1. Frequency-Domain Features

For each channel and epoch, the power spectral density (PSD) was estimated using Welch’s method. Seven frequency bands were considered: delta (1–4 Hz), theta (4–8 Hz), alpha-1 (8–10 Hz), alpha-2 (10–13 Hz), beta-1 (13–20 Hz), beta-2 (20–30 Hz), and gamma (30–45 Hz). Let $P_{c,b}$ denote the power of channel *c* in frequency band *b*. The relative power spectral density (RELPSD) was defined as

(1) $${\mathrm{RELPSD}}_{c,b}=\frac{P_{c,b}}{\sum_{j=1}^{7}P_{c,j}},$$

where *c* denotes the EEG channel, and b∈{1,…,7} denotes the frequency band. For a band-limited signal approximated by a Gaussian distribution, differential entropy (DE) was calculated as

(2) $${\mathrm{DE}}_{c,b}=\frac{1}{2}\ln\left(2\pi e\;\sigma_{c,b}^{2}\right),$$

where $\sigma_{c,b}^{2}$ is the variance of channel *c* in band *b*.

#### 3.3.2. Time-Statistical Features

To examine non-spectral identity information, eight time-statistical (TS) features were extracted independently from each EEG channel: log variance, root mean square, skewness, kurtosis, peak-to-peak amplitude, mean absolute first difference (line-length-related measure), Hjorth mobility, and Hjorth complexity. This resulted in 64 × 8 = 512 TS features per epoch. Any non-finite values arising from numerical edge cases were replaced by finite values before classification.

#### 3.3.3. Feature Sets and Temporal Training Protocols

Five feature sets were evaluated: RELPSD, DE, DE + RELPSD, TS, and DE + RELPSD + TS. The predefined closed-set primary analysis used DE + RELPSD + TS to evaluate the paradigms using a comprehensive spectral and temporal representation. The feature-comparison experiment evaluated all five feature sets under a fixed model and fixed temporal protocol.

To avoid leakage caused by randomly partitioning adjacent EEG segments, samples from each subject and paradigm were divided chronologically into five consecutive blocks. Four training–test protocols were defined according to the amount of enrollment data. P1 used B1 for training and B2–B5 for testing, representing minimal enrollment data. P2 used B1–B2 for training and B3–B5 for testing. P3 used B1–B3 for training and B4–B5 for testing. P4 used B1–B4 for training and B5 for testing, representing relatively sufficient enrollment data. These protocols allowed performance changes associated with increasing enrollment data to be quantified.

### 3.4. Machine-Learning and Deep-Learning Models

#### 3.4.1. Conventional Machine-Learning Models

Four conventional machine-learning models were compared: random forest (RF), linear discriminant analysis (LDA), extra trees (ET), and logistic regression (LR). The analysis focused on the relative strength of identity information across paradigms rather than on introducing a new classifier. RF and ET each used 200 trees and class-balanced weights. LDA used the lsqr solver with automatic shrinkage to stabilize covariance estimation in the high-dimensional feature space. LR used class-balanced weights and a maximum of 3000 iterations. For linear models requiring feature scaling, standardization parameters were fitted exclusively on the training blocks and then applied to the corresponding test blocks. Test data were not used for scaling, model fitting, or threshold selection.

The predefined closed-set primary analysis used LR to avoid selecting the best-performing classifier after observing the results. Model robustness was evaluated by comparing RF, shrinkage LDA, ET, and LR under a fixed feature set and temporal protocol. DE + LDA was retained as a fixed conventional reference pipeline for direct comparison with the additional robustness analyses. To ensure that the revised open-set conclusions did not depend on post hoc selection based on final-test performance, a separate leakage-free nested feature/classifier-selection procedure was additionally performed using only enrolled identities, as described in Section 3.6.

#### 3.4.2. Deep-Learning Baselines

Two raw-EEG deep-learning architectures were additionally evaluated: an EEGNet-FLAT model and an EEG Conformer-based model. Both models directly received minimally processed EEG epochs with 64 channels and 1000 temporal samples as input, avoiding handcrafted feature extraction. The EEGNet-FLAT architecture was adapted from the compact EEGNet framework with a modified feature aggregation strategy [17]. The network consisted of temporal convolution with eight filters ($F_{1}=8$) and a kernel length of 64 samples, followed by depthwise spatial convolution with a depth multiplier of 2 across all EEG channels. A separable convolution block generated 16 output feature maps ($F_{2}=16$). Average pooling factors of 4 and 8 and dropout (p=0.5) were applied within the feature extractor. Unlike the previous globally compressed EEGNet implementation, the EEGNet-FLAT model retained the final convolutional feature representation by flattening the learned spatiotemporal features before the identity-classification layer. This modification was introduced to avoid excessive dimensionality reduction before classification and to provide a fairer comparison with conventional feature-based pipelines. The EEG Conformer architecture consisted of a convolutional patch-embedding module followed by Transformer encoder blocks [18]. The temporal convolution kernel length was 25 samples, followed by pooling with a kernel length of 75 samples and stride of 15. The embedding dimension was 40 with 10 attention heads and a feed-forward expansion factor of 4. Both deep-learning models were trained using cross-entropy loss with a batch size of 64. EEGNet-FLAT was optimized using Adam with a learning rate of $1\times 10^{-3}$ and weight decay of $1\times 10^{-4}$, whereas EEG Conformer was optimized using AdamW with a learning rate of $2\times 10^{-4}$ and weight decay of $1\times 10^{-2}$. Channel-wise normalization parameters were estimated exclusively from the training data. No test-session data were used for model fitting, normalization, or threshold determination.

### 3.5. Closed-Set Subject-Identification Design

In the closed-set task, the training and test sets contained the same 95 identities but were drawn from non-overlapping temporal blocks. The four chronologically ordered protocols shown in Figure 1 ensured that test epochs occurred after training epochs within the recording session. This design is more appropriate than random epoch partitioning for examining short-term temporal repeatability. Prior studies have emphasized the effects of protocol design and cross-session variation on EEG-biometric evaluation [7,13]; temporal separation was, therefore, used as a control in the present paradigm comparison.

The closed-set experiments were organized as follows. Experiment 2 fixed DE + RELPSD + TS and LR and compared the five paradigms under P1–P4. Experiment 3 fixed LR and P4 and compared the five feature sets. Experiment 4 fixed DE + RELPSD + TS and P4 and compared RF, LDA, ET, and LR. Experiment 5 used the predefined primary pipeline (DE + RELPSD + TS, LR, and P4) for within-session auxiliary verification. Experiment 6 conducted statistical tests and robustness ranking across predefined analytical pipelines. Experiment 7 compared the contributions of the individual tasks within each paradigm.

As an additional model-robustness analysis, DE + LDA, EEGNet-based, and EEG Conformer-based models were compared under the P4 temporal protocol. For both deep-learning models, B1–B3 were used for initial training and B4 served as a chronologically later validation block for selecting the training epoch. The network was subsequently reinitialized and refitted using B1–B4 for the selected number of epochs, while B5 remained completely isolated for final testing. DE + LDA was trained on B1–B4 and likewise evaluated on B5. Thus, all three model families shared the same final chronological test block.

To evaluate score separation between identity matches and non-matches, multiclass outputs were also converted into within-session auxiliary verification scores. For each test epoch, the score assigned to its true identity was treated as a genuine score, whereas scores assigned to all other identities were treated as impostor scores. Equal error rate (EER), area under the receiver-operating-characteristic curve (AUC), true acceptance rate at FAR = 1% (TAR@FAR = 1%), and TAR@FAR = 0.1% were then calculated. The predefined primary statistical analysis used DE + RELPSD + TS, LR, and P4. Subject-level Rank-1 values were first aggregated over the 10 sampling repetitions. Overall differences among paradigms were tested using the Friedman test, and Kendall’s *W* was calculated as the effect-size measure. When the omnibus test was significant, pairwise Wilcoxon signed-rank tests were performed and corrected for multiple comparisons using the Holm procedure.

### 3.6. Open-Set Identification and Unknown-Identity Rejection

Closed-set identification assumes that every probe belongs to an enrolled identity and, therefore, cannot evaluate rejection of unknown users. Practical EEG biometric systems must correctly identify enrolled users while preventing unregistered identities from being assigned to enrolled classes. Recent EEG-authentication studies have increasingly emphasized standardized protocols, secure decision constraints, and realistic operating conditions [5,13].

For the within-session open-set analyses, the 95 Session 1 subjects were randomly divided into 60 enrolled identities, 15 development unknown identities, and 20 final-test unknown identities. The identity split was repeated 10 times, and the same split was used across all five paradigms within each repetition. B1–B3 from enrolled identities were used for model fitting, B4 from development unknown identities for rejection-threshold calibration, and B5 from enrolled and final-test unknown identities for final evaluation.

The original DE + LDA configuration was retained as a fixed conventional reference. To exclude potential identity leakage during data-dependent model selection, an additional nested procedure selected among five feature representations (RELPSD, DE, DE + RELPSD, TS, and DE + RELPSD + TS) and four conventional classifiers (RF, shrinkage LDA, ET, and LR) using enrolled identities only. Candidate models were trained on B1–B2 and validated on B3, and a single feature–classifier combination was selected per repetition according to mean validation Rank-1 accuracy across the five paradigms. The selected model was then refitted on B1–B3 before threshold calibration and final testing. Neither development nor final-test unknown identities were used for feature or classifier selection.

EEGNet-based and EEG Conformer-based models were additionally evaluated using the same 60/15/20 identity partitions. For the deep models, B1–B2 were used for training, B3 for temporal validation, B4 development unknown probes for threshold calibration, and B5 for final testing. Maximum LDA decision score and maximum deep-model logit were used as the primary confidence scores. Open-set performance was quantified using DIR at FPIR = 5% and 1%, realized final-test FPIR, AU-OSCR, unknown-detection AUROC, and AUPR. Paradigm comparisons of DIR used subject-level Friedman tests followed by Holm-corrected Wilcoxon signed-rank tests when appropriate.

### 3.7. Cross-Session Generalization Analysis

To evaluate whether within-session identity-discriminative information generalized to an independently recorded session, an additional Session 1-to-Session 2 analysis was performed using the labeled Testing data as Session 2. Seventy-six subjects satisfied all task-specific quotas across the five paradigms and were eligible for cross-session analysis. In each repetition, the 76 eligible subjects were divided into 60 enrolled identities, 8 development unknown identities, and 8 final-test unknown identities. For the primary source-only condition, models were trained exclusively on Session 1 and evaluated directly on task-matched Session 2 probes. DE + LDA was trained on B1–B4, whereas EEGNet-based and EEG Conformer-based models used B1–B3 for training, B4 for temporal validation, and were subsequently refitted on B1–B4. No Session 2 identity labels were used for model fitting or supervised parameter selection. A same-pipeline within-session control evaluated the corresponding Session 1-trained models on held-out Session 1 B5 probes using the same identity splits and scoring procedures. This control was used to distinguish cross-session degradation from implementation-related failure. An additional target-normalization sensitivity analysis estimated normalization statistics from pooled unlabeled Session 2 probes while leaving the Session 1-trained classifier unchanged. Because this analysis observed the target-session feature distribution, it was treated as a diagnostic sensitivity analysis rather than as the primary zero-shot condition. An additional target-session threshold-calibration analysis was likewise considered diagnostic and was not interpreted as zero-shot cross-session performance.

### 3.8. Implementation and Reproducibility

All analyses were implemented in Python 3.8.20 using NumPy 1.24.4, SciPy 1.10.1, scikit-learn 1.3.2, and PyTorch 2.4.1. Predetermined random seeds were used for repeated sampling and identity partitioning, and the same repeat-specific splits were reused across paradigms whenever paired comparisons were required. For deep-learning experiments, deterministic execution was enabled where supported. The cross-session implementation additionally verified consistency among SubjectID, EpochID, feature arrays, and the task-matched Session 2 sampling manifest before model evaluation.

## 4. Results

### 4.1. Closed-Set Identification Under the Temporally Ordered Protocols

**Figure 2:**
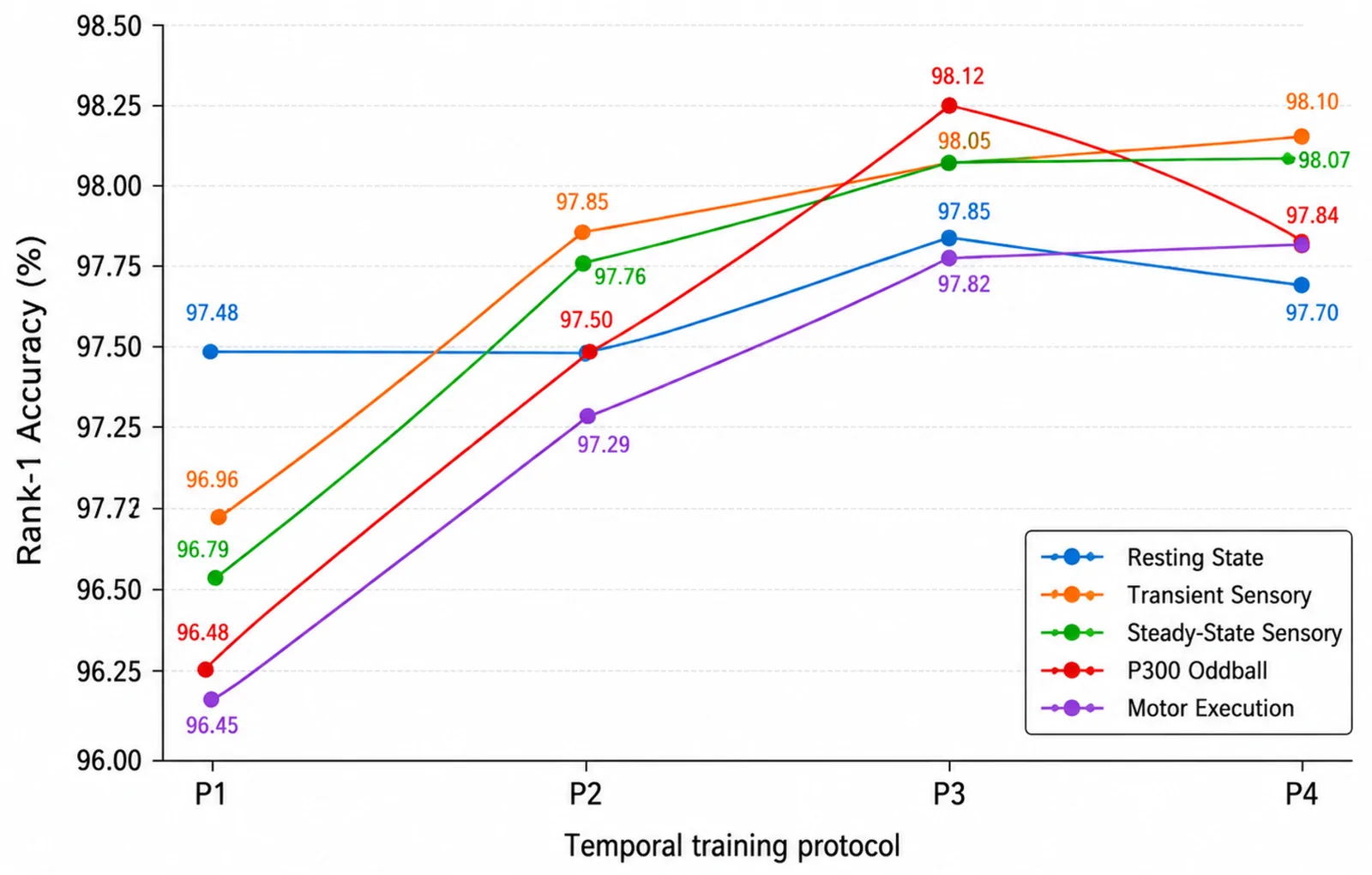
Rank-1 accuracy of the five paradigms under the P1–P4 temporally ordered training–test protocols.

With DE + RELPSD + TS and LR fixed, the five paradigms were compared under P1–P4 (Table 1 and Figure 2). Under P1, in which only B1 was used for training and B2–B5 were used for testing, Rank-1 accuracy exceeded 95% for every paradigm. Transient sensory stimulation achieved the highest P1 Rank-1 accuracy (96.96%), whereas motor execution achieved the lowest (95.80%). Performance generally increased as the number of training blocks increased.

Under P2, transient and steady-state sensory stimulation achieved Rank-1 accuracies of 97.85% and 97.79%, respectively. Under P3, P300 Oddball achieved the highest Rank-1 accuracy (98.11%), although the differences from transient and steady-state sensory stimulation were small. Under the predefined P4 primary protocol (B1–B4 training and B5 testing), transient sensory stimulation achieved the highest Rank-1 accuracy (98.10%). From P1 to P4, the largest increase was observed for motor execution, which improved from 95.80% to 97.86% (2.05 percentage points). The corresponding increases for resting state, P300 Oddball, steady-state sensory stimulation, and transient sensory stimulation were 1.60, 1.57, 1.27, and 1.14 percentage points, respectively. These results indicate that transient sensory stimulation performed strongly even with limited enrollment data, whereas motor execution was more sensitive to the amount of training data.

### 4.2. Effect of Feature Representation

With LR and P4 fixed, RELPSD, DE, DE + RELPSD, TS, and DE + RELPSD + TS were compared (Table 2A and Supplementary Figure S1A. Averaged across the five paradigms, DE achieved the highest Rank-1 accuracy (98.01%), the lowest mean EER (0.187%), and a TAR@FAR = 1% of 99.91%. DE + RELPSD achieved a mean Rank-1 accuracy of 97.95%, close to but not exceeding DE. The DE + RELPSD + TS representation used in the predefined primary analysis achieved a mean Rank-1 accuracy of 97.92% and a mean EER of 0.313%. RELPSD and TS alone yielded lower mean Rank-1 accuracies of 96.25% and 95.33%, respectively.

DE achieved the highest or near-highest Rank-1 accuracy for resting state, transient sensory stimulation, P300 Oddball, and motor execution. Transient sensory stimulation reached 98.27% with DE, the highest value in the feature comparison. Steady-state sensory stimulation achieved a slightly higher value with DE + RELPSD + TS (98.08%). Overall, DE effectively represented subject-specific differences under within-session temporal separation, whereas adding RELPSD or time-statistical features did not produce a consistent improvement. The relatively weak performance of TS alone suggests that simple temporal summary statistics were insufficient to characterize the identity information in this setting.

### 4.3. Comparison of Machine-Learning Models

With DE + RELPSD + TS and P4 fixed, RF, shrinkage LDA, ET, and LR were compared (Table 2B and Supplementary Figure S1B. Averaged across paradigms, LDA achieved the best overall performance, with a Rank-1 accuracy of 98.29%, EER of 0.073%, AUC of 99.98%, and TAR@FAR = 1% of 99.94%. LR achieved a mean Rank-1 accuracy of 97.92%, whereas ET and RF achieved 96.87% and 96.81%, respectively.

LDA achieved the highest Rank-1 accuracy in every paradigm. Under LDA, transient sensory stimulation reached 98.60%, the highest value in the model comparison; motor execution, P300 Oddball, steady-state sensory stimulation, and resting state achieved 98.32%, 98.28%, 98.18%, and 98.07%, respectively. These findings indicate that identity-related differences were strongly linearly separable in the evaluated feature space and that shrinkage estimation was well suited to the high-dimensional EEG representation. Although LR was retained as the predefined primary model to avoid post hoc model selection, LDA provided the strongest robustness result.

### 4.4. Within-Session Auxiliary Verification Performance

Under the predefined primary pipeline (DE + RELPSD + TS, LR, and P4), EER, AUC, TAR@FAR = 1%, and TAR@FAR = 0.1% were calculated Supplementary Table S1. Transient sensory stimulation achieved the highest Rank-1 accuracy (98.10%; 95% CI, 96.12–99.50%), and steady-state sensory stimulation achieved 98.08% (95% CI, 95.48–99.83%). The Rank-1 confidence intervals overlapped substantially, indicating near-saturated closed-set identification performance.

Steady-state sensory stimulation achieved the lowest EER (0.244%), an AUC of 99.99%, and the highest TAR@FAR=1% (99.97%). EER values for resting state, P300 Oddball, transient sensory stimulation, and motor execution were 0.249%, 0.303%, 0.312%, and 0.457%, respectively. At FAR = 0.1%, transient sensory stimulation achieved the highest TAR (99.18%), followed by P300 Oddball (98.87%), steady-state sensory stimulation (98.82%), motor execution (98.81%), and resting state (98.25%). Thus, steady-state sensory stimulation showed strong closed-set score separation at EER and TAR@FAR = 1%, whereas transient sensory stimulation showed advantages in Rank-1 and TAR@FAR = 0.1%.

### 4.5. Statistical Testing and Robustness of Closed-Set Identification

Under DE + RELPSD + TS, LR, and P4, the omnibus subject-level Rank-1 difference among paradigms was statistically significant (Friedman $\chi^{2}=20.362$, p=0.000424; Supplementary Table S2). However, Kendall’s *W* was only 0.0536, indicating a small effect. After pairwise Wilcoxon signed-rank tests with Holm correction, no pair of paradigms differed significantly. Thus, despite a significant omnibus difference, the practical differences were small because all paradigms performed near the ceiling.

Robustness rankings across all predefined conventional analytical pipelines are shown in Supplementary Figure S2A and Supplementary Table S2. Transient sensory stimulation had the lowest mean rank (1.45) and ranked first in 7 of the 11 pipelines. Steady-state sensory stimulation ranked second on average (2.55) and was first in 3 pipelines. P300 Oddball, resting state, and motor execution had mean ranks of 3.18, 3.82, and 4.00, respectively. These results indicate that transient sensory stimulation provided the most consistent numerical advantage across the predefined conventional feature, classifier, and temporal-protocol analyses.

### 4.6. Within-Paradigm Contributions of Individual Tasks

The individual tasks within each paradigm were compared under DE + RELPSD + TS, LR, and P4 (Supplementary Figure S2B). In the resting-state paradigm, Task 1 (eyes-open resting state) and Task 2 (eyes-closed resting state) achieved Rank-1 accuracies of 96.84% and 97.37%, respectively; the difference was not significant. In transient sensory stimulation, Task 3 (transient visual stimulation), Task 4 (transient auditory stimulation), and Task 5 (transient audiovisual stimulation) achieved 97.33%, 97.47%, and 97.46%, respectively. The differences were not significant (p=0.319, Kendall’s W=0.012), indicating that the paradigm-level advantage was not driven by a single task.

In steady-state sensory stimulation, Task 6 (steady-state visual stimulation), Task 7 (steady-state auditory stimulation), and Task 8 (steady-state audiovisual stimulation) achieved 97.54%, 97.93%, and 97.65%, respectively. Although Task 7 was numerically highest, the omnibus difference was not significant (p=0.076, Kendall’s W=0.027). In P300 Oddball, Task 9 (visual Oddball) and Task 10 (auditory Oddball) achieved 98.15% and 97.43%; Task 9 remained significantly better after Holm correction (p<0.0001). In motor execution, Task 11 (left-hand movement), Task 12 (right-hand movement), and Task 13 (bilateral foot movement) achieved 96.95%, 96.68%, and 97.47%, respectively. The omnibus difference was significant (p<0.0001, Kendall’s W=0.098), and Task 13 significantly outperformed Tasks 11 and 12. Thus, transient and steady-state sensory stimulation showed relatively consistent task-level performance, whereas P300 Oddball and motor execution showed stronger task dependence.

### 4.7. Leakage-Free Open-Set Identification and Unknown-Identity Rejection

The open-set analysis was repeated using the identity-first nested selection procedure to determine whether the findings depended on data-dependent feature or classifier selection. Across the 10 repetitions, shrinkage LDA was selected in all repetitions; DE was selected in eight repetitions, whereas DE + RELPSD was selected in the remaining two. Thus, the nested procedure consistently favored a low-complexity spectral representation combined with a shrinkage linear classifier without using development or final-test unknown identities for feature or classifier selection. When rejection was disabled, enrolled-only Rank-1 accuracy remained uniformly high across the five paradigms, ranging from 98.03% to 98.50%. At the target FPIR of 5%, transient sensory stimulation achieved the highest DIR (91.75 ± 12.49%), followed by P300 Oddball (88.92 ± 22.20%), steady-state sensory stimulation (88.15 ± 20.27%), motor execution (84.28 ± 22.59%), and resting state (80.85 ± 23.36%). The realized FPIR in the independent final-test unknown set ranged from 5.79% to 6.92% (Table 3).

At the stricter target FPIR of 1%, transient sensory stimulation again achieved the highest DIR (77.32 ± 25.81%), followed by P300 Oddball (74.22 ± 23.03%), motor execution (58.15 ± 31.27%), steady-state sensory stimulation (52.74 ± 34.91%), and resting state (51.68 ± 27.49%). The realized final-test FPIR ranged from 2.58% to 3.79%. Threshold-independent evaluation showed a similar numerical pattern. Transient sensory stimulation achieved the highest AU-OSCR (97.36 ± 0.43%), together with an unknown-identity AUROC of 98.74 ± 0.84% and AUPR of 97.66 ± 1.13%. AU-OSCR values for the other paradigms ranged from 96.34% to 96.67%. These results show that the favorable within-session open-set performance of transient sensory stimulation persisted after feature and classifier selection was restricted entirely to enrolled identities.

### 4.8. Comparison Between Closed-Set Identification and Open-Set Rejection

**Figure 3:**
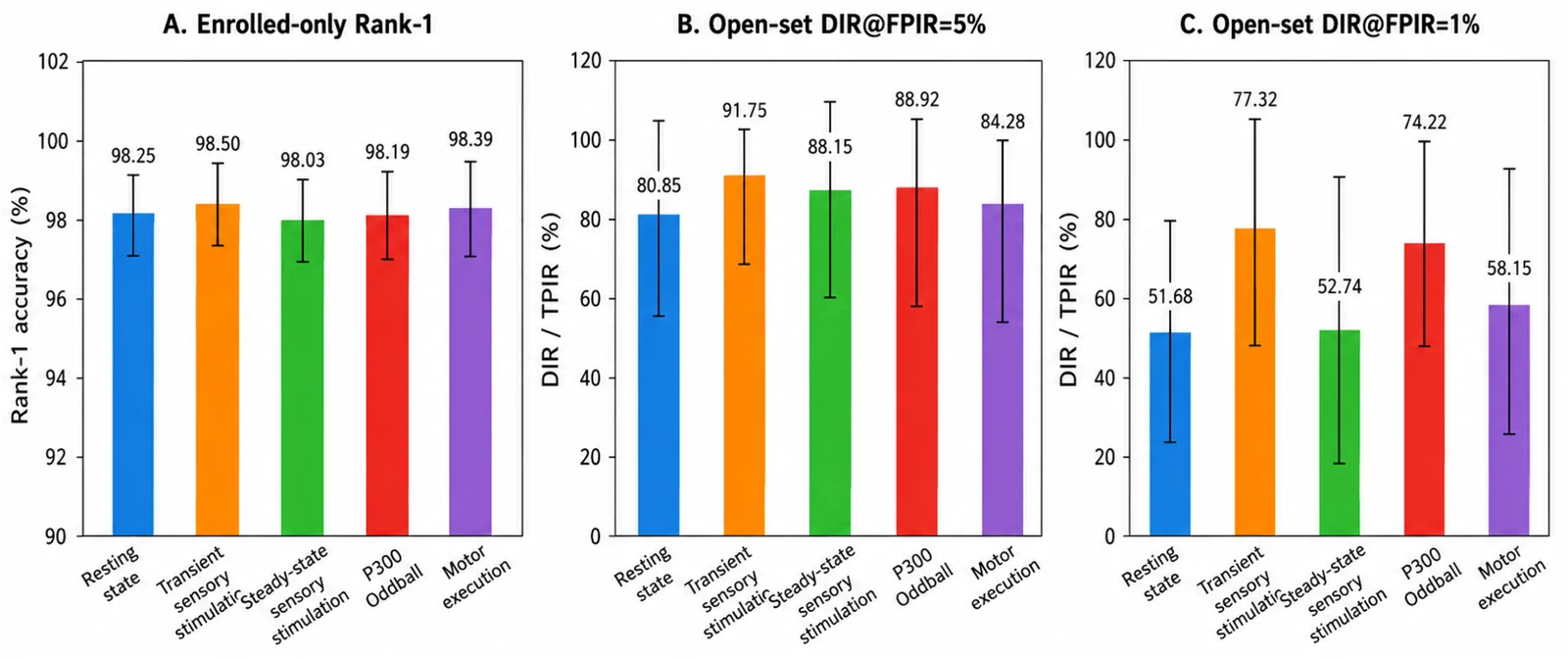
Leakage-Free Open-Set Identification and Rejection Performance across Paradigms.

The leakage-free nested experiment further demonstrated the difference between discrimination among enrolled identities and identification under unknown-user rejection constraints (Figure 3). Enrolled-only Rank-1 accuracy varied by only 0.47 percentage points across paradigms, from 98.03% to 98.50%. In contrast, DIR at FPIR = 5% ranged from 80.85% to 91.75%, and DIR at FPIR = 1% ranged from 51.68% to 77.32%. For transient sensory stimulation, performance decreased from 98.50% enrolled-only Rank-1 accuracy to 91.75% DIR at FPIR = 5% and 77.32% at FPIR = 1%. The separation among paradigms, therefore, became substantially larger as the evaluation moved from classification among enrolled identities to unknown-identity rejection, particularly at the stricter FPIR = 1% operating point.

### 4.9. Statistical Comparison of Open-Set DIR

**Figure 4:**
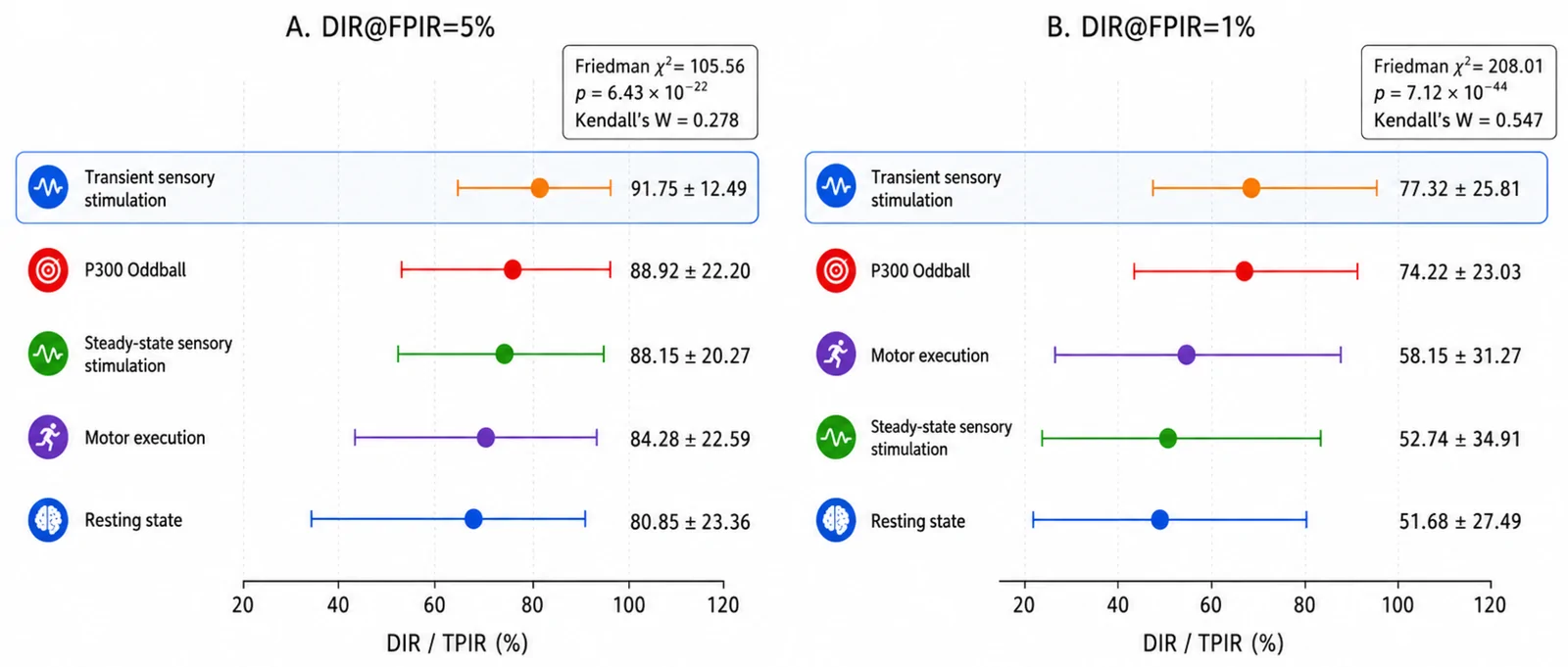
Open-set DIR and subject-level inferential statistics across paradigms.

Subject-level analyses confirmed significant paradigm effects under the leakage-free nested protocol (Figure 4; Table 3; Supplementary Table S5). At FPIR = 5%, DIR differed significantly among the five paradigms (Friedman $\chi^{2}=105.56$, $p=6.43\times 10^{-22}$; Kendall’s W=0.278). After Holm correction across all pairwise paradigm comparisons, transient sensory stimulation significantly outperformed resting state, steady-state sensory stimulation, P300 Oddball, and motor execution (all adjusted p<0.001). At FPIR = 1%, the paradigm effect became substantially stronger (Friedman $\chi^{2}=208.01$, $p=7.12\times 10^{-44}$; Kendall’s W=0.547). Transient sensory stimulation significantly outperformed resting state, steady-state sensory stimulation, and motor execution after Holm correction. Its numerical advantage over P300 Oddball did not remain significant after correction across all 10 pairwise comparisons ($p_{\mathrm{Holm}}=0.0752$). The increase in Kendall’s *W* from 0.278 to 0.547 indicates that paradigm-related differences became more pronounced under the stricter rejection constraint.

### 4.10. Robustness Across Conventional and Deep-Learning Models

**Figure 5:**
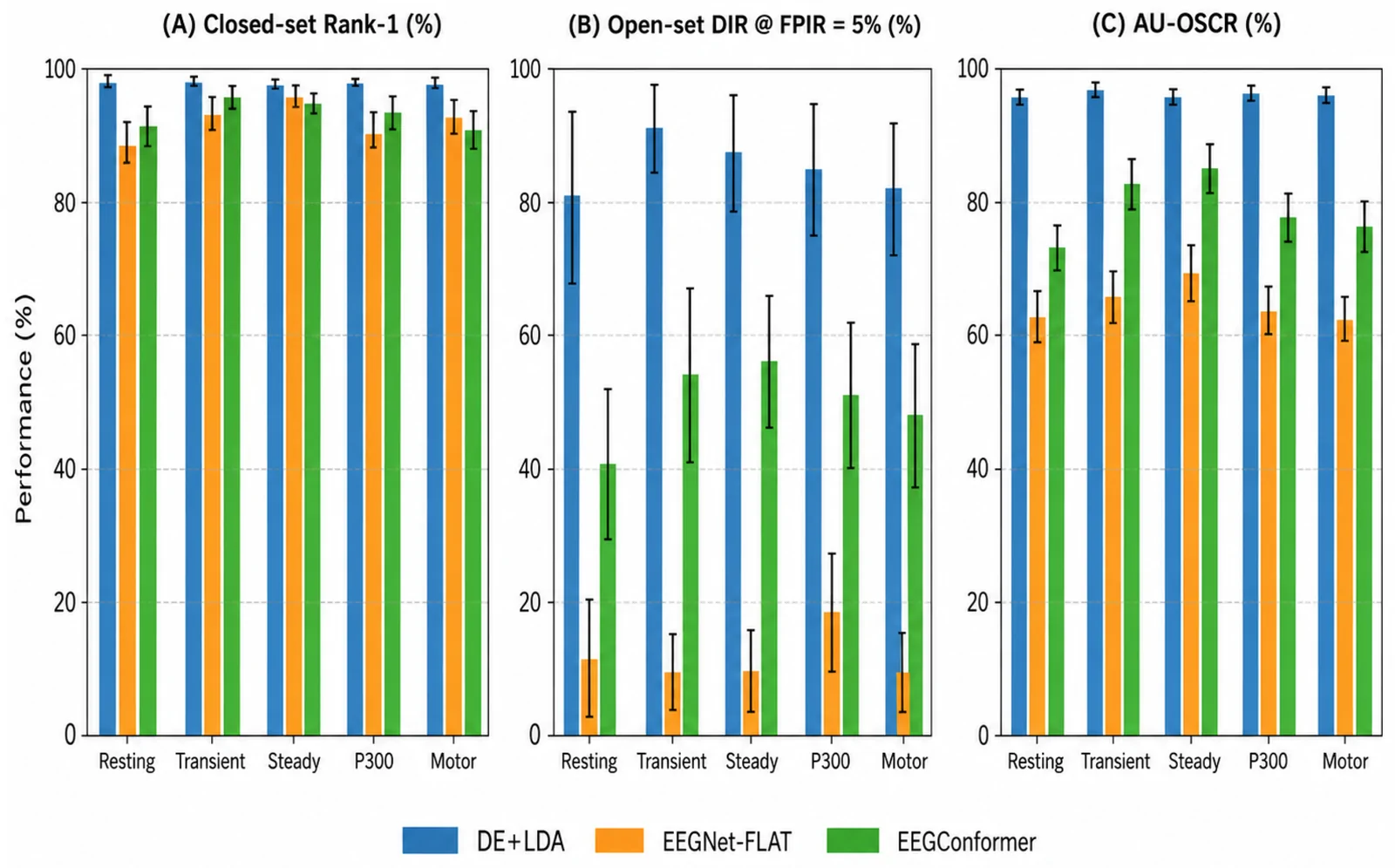
Robustness across Conventional and Deep-Learning Model Families.

To evaluate whether the observed identity discrimination was specific to handcrafted spectral representations, DE + LDA was compared with EEGNet-FLAT and EEG Conformer under the matched within-session P4 temporal protocol (Figure 5; Supplementary Table S3). DE + LDA achieved consistently high closed-set identification performance, with Rank-1 accuracies above 98% across all five paradigms. EEGNet-FLAT achieved Rank-1 accuracies ranging from 88.87% to 96.22%, whereas EEG Conformer achieved intermediate performance ranging from 91.08% to 96.03%. Compared with the previous EEGNet implementation, EEGNet-FLAT substantially improved within-session identification performance. The Rank-1 accuracy increased by 9.21 percentage points for resting state, 1.73 percentage points for transient sensory stimulation, 5.73 percentage points for steady-state sensory stimulation, 10.09 percentage points for P300 Oddball, and 9.76 percentage points for motor execution. These improvements indicate that excessive feature compression before classification can substantially reduce EEG biometric performance. Despite the improvement of EEGNet-FLAT, DE + LDA remained the strongest overall closed-set identification pipeline. This result suggests that frequency-domain statistical representations combined with shrinkage linear discrimination remain highly effective for high-dimensional EEG identity classification under the current dataset scale. Open-set rejection showed stronger model dependence. EEGNet-FLAT achieved lower DIR@FPIR = 5% and AU-OSCR values than DE + LDA and EEG Conformer, indicating that strong closed-set identification accuracy does not necessarily translate into reliable unknown-identity rejection. These findings demonstrate that deep-learning models can capture discriminative EEG identity information, while conventional feature-based approaches remain competitive when evaluated under strict open-set biometric conditions.

### 4.11. Zero-Shot Cross-Session Generalization

**Figure 6:**
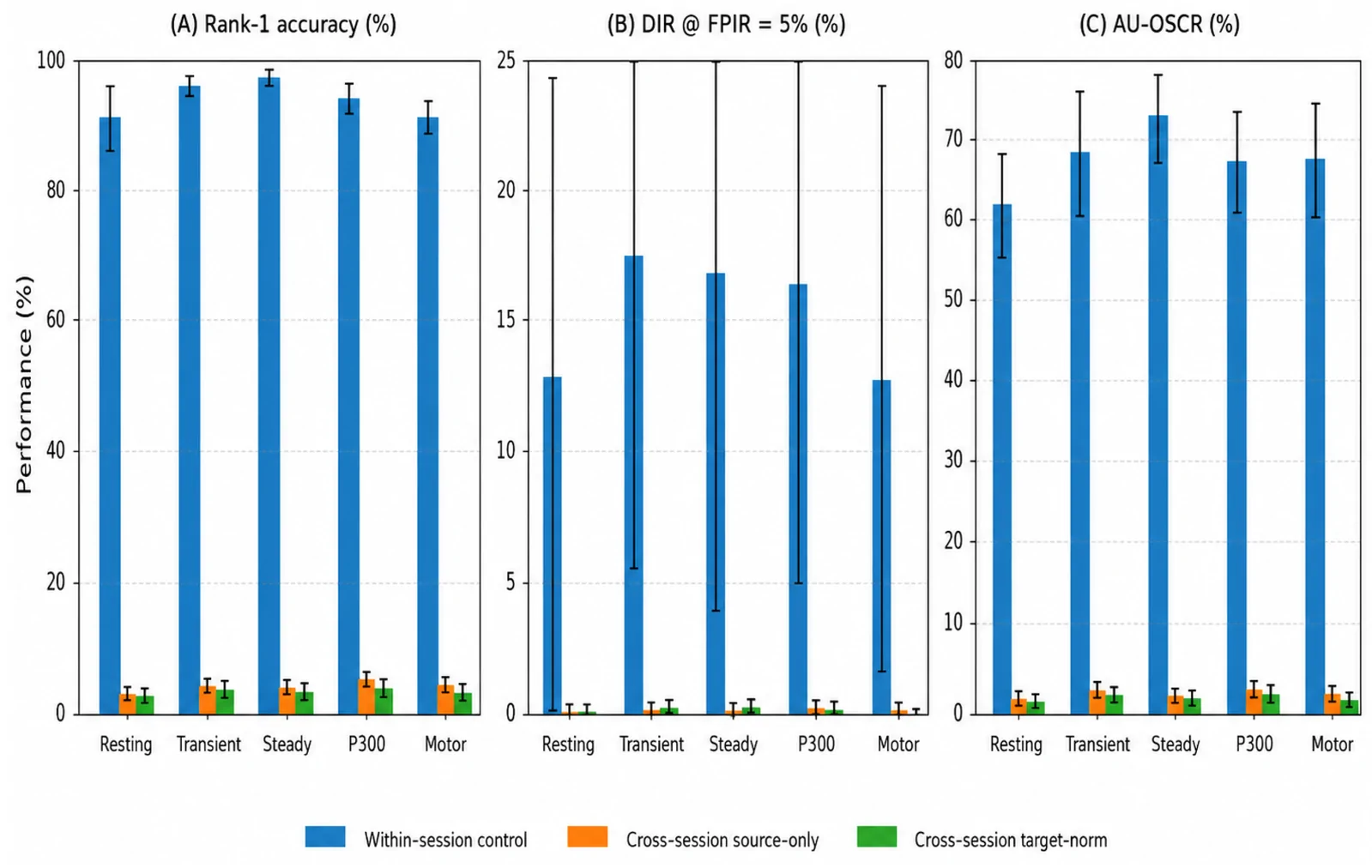
Zero-Shot Cross-Session Generalization and Target-Normalization Sensitivity.

Cross-session generalization of EEGNet-FLAT was evaluated using the E10-v2 protocol, where models were trained on Session 1 and directly tested on Session 2 without using Session 2 identity labels. A within-session control condition and an unsupervised target-normalization diagnostic were additionally included (Figure 6; Supplementary Table S4). Under the within-session control condition, EEGNet-FLAT achieved Rank-1 accuracies ranging from 90.93% to 97.86% across the five paradigms, confirming that the revised architecture retained strong identity-discriminative capability when training and testing data were acquired within the same recording session. However, strict source-only cross-session transfer resulted in severe performance degradation. Rank-1 accuracy decreased to 2.36–4.21% across paradigms, approaching the chance level for the 76-subject cross-session cohort. Target normalization using unlabeled Session 2 data provided no meaningful recovery, with performance remaining close to chance level. Open-set rejection showed the same pattern. Under within-session evaluation, EEGNet-FLAT achieved DIR@FPIR = 5% values of 12.65–16.93% and AU-OSCR values of 61.99–72.69%. In contrast, source-only cross-session transfer reduced DIR@FPIR=5% to below 0.25% and AU-OSCR to approximately 1–2%. These results indicate that EEGNet-FLAT successfully learns session-specific identity representations but remains highly sensitive to inter-session variability. Simple distribution normalization was insufficient to overcome the session-dependent shift, highlighting cross-session stability as a major challenge for practical EEG biometric deployment.

## 5. Discussion

This study compared the resting state, transient sensory stimulation, steady-state sensory stimulation, P300 Oddball, and motor execution under a common sample budget and temporally ordered evaluation framework. All five paradigms showed strong within-session identity discriminability, with P4 Rank-1 accuracies close to 98% even though training and test blocks were strictly separated in time. This finding is consistent with recent EEG-biometric studies showing that subject-specific frequency structure, evoked responses, and task-related patterns can support identity discrimination [19,20,21].

The within-session closed-set results nevertheless showed a clear ceiling effect. Under P4, the difference between the highest and lowest Rank-1 accuracy was only 0.38 percentage points, and the confidence intervals overlapped considerably. Although the omnibus Friedman test was significant, the effect size was small, and no pairwise comparison remained significant after Holm correction. Therefore, within-session closed-set Rank-1 accuracy alone provided limited discrimination among paradigms when overall performance approached saturation. Previous work has similarly shown that EEG-biometric performance can be strongly affected by time-window selection, data partitioning, and recording state [22]. The present results further demonstrate that high within-session accuracy should be interpreted as evidence of identity discriminability under a particular recording condition rather than as proof of session-invariant biometric stability or secure open-set operation.

Across the predefined conventional analytical pipelines, transient sensory stimulation showed the most stable numerical advantage: it ranked first in 7 of 11 pipelines and had the lowest average rank. The leakage-free nested open-set analysis supported the same overall within-session pattern, with transient sensory stimulation achieving the highest numerical DIR at both predefined FPIR operating points. Within-paradigm task analysis further showed no significant differences among transient visual, auditory, and audiovisual stimulation, indicating that this advantage was not driven by one isolated task. Transient stimulation provides an explicit temporal anchor for sensory and cognitive processing and may be less susceptible than resting state to unconstrained state fluctuations. It may also reduce the fatigue and sustained-attention variability associated with continuous stimulation. Recent studies using visual-evoked, auditory-evoked, and event-related EEG have likewise demonstrated that externally evoked responses can provide repeatable and discriminative identity information [23,24,25]. However, the additional deep-learning and cross-session analyses showed that this within-session advantage should not be interpreted as universal superiority of transient sensory stimulation across model families or recording sessions.

A central finding was that leakage-free open-set evaluation revealed security-relevant differences that were largely hidden by closed-set metrics. Although enrolled-only Rank-1 accuracy remained between 98.03% and 98.50% across paradigms, DIR at FPIR = 1% ranged from 51.68% to 77.32%. Transient sensory stimulation achieved the highest numerical DIR at both FPIR = 5% (91.75%) and FPIR = 1% (77.32%), whereas substantially larger performance losses were observed for several other paradigms as the rejection constraint was tightened. Importantly, these findings were obtained after feature and classifier selection had been restricted to enrolled identities, with development unknown identities used only for threshold calibration and final-test unknown identities reserved for final evaluation. The observed paradigm differences, therefore, cannot be attributed to using final-test unknown identities during feature or classifier selection. High closed-set accuracy, therefore, did not guarantee effective rejection of unregistered identities. Practical authentication must correctly identify enrolled users while avoiding forced assignment of unknown users to enrolled classes. EEG-authentication research has increasingly emphasized security threats, robust authentication, and explicit rejection mechanisms [26,27]; the present results further support the routine reporting of DIR, FPIR, and threshold-independent open-set metrics in EEG biometric evaluation.

Steady-state sensory stimulation performed strongly in closed-set auxiliary verification, particularly in EER and TAR@FAR = 1%, but did not retain this advantage under strict open-set conditions. This divergence indicates that separation between genuine and impostor scores derived from enrolled identities is not equivalent to rejection of previously unseen identities. In the leakage-free nested analysis, transient sensory stimulation achieved the highest numerical DIR, AU-OSCR, unknown-identity AUROC, and AUPR. Subject-level testing confirmed significant overall paradigm effects at both operating points, and Kendall’s *W* increased from 0.278 at FPIR = 5% to 0.547 at FPIR = 1%, indicating greater between-paradigm differentiation under the stricter rejection requirement. At FPIR = 5%, transient sensory stimulation significantly exceeded all four other paradigms after Holm correction. At FPIR = 1%, it significantly exceeded resting state, steady-state sensory stimulation, and motor execution, whereas its numerical advantage over P300 Oddball did not remain significant after correction across all 10 pairwise comparisons ($p_{\mathrm{Holm}}=0.0752$). Thus, the results support a strong within-session open-set advantage for transient sensory stimulation relative to several competing paradigms but do not justify treating every numerical difference as statistically distinct.

The feature and model comparisons showed that DE and shrinkage LDA were well suited to the present data. DE achieved the highest average Rank-1 and the lowest average EER among the five feature sets, suggesting that frequency-band energy distributions expressed in entropy form capture stable individual differences. LDA outperformed RF, ET, and LR with the fixed fused representation, indicating strong linear separability in the resulting feature space. Time–frequency, spatial–frequency, and functional-connectivity representations have also been shown to encode EEG identity information effectively [20,28]. The additional raw-EEG analyses further showed that the paradigm comparison was not restricted to handcrafted features. Under the present training protocol, EEG Conformer consistently achieved higher closed-set Rank-1 accuracy than EEGNet, whereas DE+LDA remained strongest overall. Both deep-learning models nevertheless achieved their highest closed-set Rank-1 accuracy under transient sensory stimulation, supporting the presence of strong within-session identity information in this paradigm across different representation families. In contrast, open-set performance was substantially more model-dependent. EEG Conformer clearly exceeded EEGNet, but its highest DIR and AU-OSCR occurred under steady-state rather than transient sensory stimulation. These findings should not be interpreted as evidence that conventional models are intrinsically superior to deep learning; rather, they indicate that architecture, training regime, learned representation, and confidence-score behavior can materially affect open-set conclusions. The use of conventional features and classifiers in the present work was intended to minimize confounding by model complexity rather than to discount deep learning. Graph neural networks, domain-adaptation networks, and other representation-learning methods remain promising for cross-session generalization, multimodal fusion, and online deployment [23,25,29].

The strict Session 1-to-Session 2 analysis exposed a substantially more severe limitation than was apparent from the within-session experiments. DE + LDA maintained approximately 99.7–100% Rank-1 accuracy in the same-pipeline within-session control, yet its accuracy decreased to only 4.49–5.40% under source-only Session 1-to-Session 2 transfer. EEG Conformer and EEGNet showed the same qualitative degradation, reaching only 2.90–3.82% and 1.93–3.33%, respectively. Verification AUC also decreased to approximately 0.53–0.56, showing that the loss was not restricted to multiclass identification but also affected genuine–impostor score separability. The same-pipeline Session 1 control remained highly effective, making a general implementation failure an unlikely explanation for the cross-session collapse. Instead, the results identify session dependence as a major limitation of the identity representations evaluated here.

Simple normalization was insufficient to resolve this problem. Unsupervised target normalization changed DE + LDA Rank-1 accuracy only marginally and produced no systematic recovery for the deep-learning models; open-set rejection also remained very low under cross-session transfer. Moreover, the marginal DE distribution-shift diagnostics showed only modest changes in global feature means and variances despite the severe loss of biometric performance. This pattern suggests that the session effect cannot be explained solely by a simple global shift in feature location or scale and may instead involve disruption of subject-specific or class-conditional structure. This interpretation remains inferential because the present analysis did not directly quantify identity-specific representation drift or class-conditional manifold alignment. Importantly, the small numerical differences among paradigms were not consistent across model families in Session 2; therefore, the cross-session results do not support identification of a single best paradigm under zero-shot transfer.

Taken together, the findings can be interpreted in terms of three related but distinct properties of EEG identity representation: within-session identity stability, enrolled–unknown separation, and cross-session invariance. Within-session identity stability describes the consistency of subject-specific patterns across temporally separated segments from the same recording session and primarily supports closed-set identification. Enrolled–unknown separation describes the stability of the decision boundary when previously unseen identities are introduced and determines open-set rejection performance. Cross-session invariance requires subject-specific information to remain sufficiently stable after changes in recording session and associated physiological or acquisition conditions. The present results show that strong performance at the first level does not guarantee performance at the second, and neither guarantees the third. Transient sensory stimulation provided comparatively favorable within-session open-set separation under the primary conventional framework, but this advantage did not persist as a stable cross-session ranking across model families. Practical EEG biometric evaluation should, therefore, distinguish explicitly between within-session discrimination, unknown-identity rejection, and true session-to-session generalization rather than treating them as interchangeable measures of biometric robustness.

Several limitations should be acknowledged. First, although the independent Session 2 data provided a stricter cross-session test, all analyses were conducted within the M3CV dataset and, therefore, do not constitute external validation across different cohorts, devices, or recording protocols. The cross-session analysis was further limited to 76 eligible subjects and two recording sessions, so longer-term biometric stability remains unknown. In addition, the target-normalization analysis was transductive and diagnostic because unlabeled target-session data were used to estimate normalization statistics and should not be interpreted as strict zero-shot deployment. Second, the unknown-identity cohorts were relatively small, particularly in the cross-session experiment, which may reduce the stability of threshold estimation at low FPIR. EEGNet and EEG Conformer were also intended as representative deep-learning baselines rather than an exhaustive comparison of architectures, domain-adaptation methods, or open-set strategies. Low-channel, dry-electrode, ear-EEG, and mobile deployment conditions were not evaluated; practical systems must also consider acquisition burden, comfort, and device usability [21,30]. Finally, EEG may reveal sensitive information about emotion, cognitive load, and health. Future systems should, therefore, combine recognition performance with template protection, privacy-preserving representations, and adversarial-attack defenses [27,31].

## 6. Conclusions

This study systematically compared five EEG experimental paradigms for subject identification and unknown-identity rejection under a unified sample budget and temporally ordered evaluation framework. All paradigms showed strong within-session identity discriminability, while transient sensory stimulation provided the most consistent advantage under the primary conventional within-session analyses and leakage-free open-set evaluation. The additional deep-learning experiments showed that open-set paradigm rankings were partly model dependent, indicating that conclusions based on a single representation or classifier should be interpreted cautiously. More importantly, strict zero-shot cross-session evaluation revealed substantial degradation across all paradigms and model families, and simple target normalization did not restore within-session performance. These findings demonstrate that within-session identity discrimination, unknown-identity rejection, and cross-session invariance represent distinct requirements for EEG biometrics. Future work should, therefore, prioritize session-robust representation learning, larger and more heterogeneous unknown-identity cohorts, independent external validation, and practical acquisition settings when assessing the real-world viability of EEG-based biometric systems.

## Figures and Tables

**Table 1:** Closed-set identification results under the four temporally ordered enrollment protocols. Values are mean ± standard deviation (%).

Paradigm	P1: B1 → B2–B5	P2: B1–B2 → B3–B5	P3: B1–B3 → B4–B5	P4: B1–B4 → B5	P4–P1
Resting state	96.12 ± 0.00	97.49 ± 0.00	97.85 ± 0.00	97.72 ± 0.00	+1.60
Transient sensory stimulation	96.96 ± 0.22	97.85 ± 0.16	98.05 ± 0.19	98.10 ± 0.33	+1.14
Steady-state sensory stimulation	96.80 ± 0.19	97.79 ± 0.07	98.06 ± 0.12	98.08 ± 0.15	+1.27
P300 Oddball	96.29 ± 0.35	97.54 ± 0.21	98.11 ± 0.16	97.86 ± 0.29	+1.57
Motor execution	95.80 ± 0.37	97.36 ± 0.25	97.84 ± 0.29	97.86 ± 0.38	+2.05

**Table 2:** Comparison of feature representations and machine-learning models.

**(A) Feature comparison with LR fixed; values are averages across the five paradigms.**					
**Feature set**	**Dimension**	**Rank-1 (%)**	**EER (%)**	**AUC (%)**	**TAR@FAR = 1% (%)**
RELPSD	448	96.25	0.616	99.95	99.55
DE	448	98.01	0.187	99.97	99.91
DE + RELPSD	896	97.95	0.255	99.97	99.89
TS	512	95.33	0.899	99.77	99.15
DE + RELPSD + TS	1408	97.92	0.313	99.95	99.84
**(B) Model comparison with DE + RELPSD + TS fixed; values are averages across the five paradigms.**					
**Model**		**Rank-1 (%)**	**EER (%)**	**AUC (%)**	**TAR@FAR = 1% (%)**
Random forest (RF)		96.81	0.592	99.92	99.56
Shrinkage LDA		98.29	0.073	99.98	99.94
Extra trees (ET)		96.87	1.456	99.17	98.43
Logistic regression (LR)		97.92	0.313	99.95	99.84

**Table 3:** Leakage-free nested open-set identification and unknown-identity rejection results.

**(A) Open-set performance at fixed FPIR operating points.**					
**Paradigm**	**Enrolled Rank-1 (%)**	**DIR@5% (%)**	**Final FPIR (%)**	**DIR@1% (%)**	**Final FPIR (%)**
Resting state	98.25 ± 1.21	80.85 ± 23.36	6.00 ± 5.05	51.68 ± 27.49	2.71 ± 3.03
Transient sensory stimulation	98.50 ± 0.93	91.75 ± 12.49	6.92 ± 4.70	77.32 ± 25.81	3.79 ± 3.70
Steady-state sensory stimulation	98.03 ± 1.19	88.15 ± 20.27	5.96 ± 3.89	52.74 ± 34.91	2.92 ± 3.79
P300 Oddball	98.19 ± 1.16	88.92 ± 22.20	5.79 ± 4.44	74.22 ± 23.03	3.21 ± 2.76
Motor execution	98.39 ± 1.07	84.28 ± 22.59	6.08 ± 4.45	58.15 ± 31.27	2.58 ± 2.96
**(B) Threshold-independent open-set metrics.**					
**Paradigm**	**AU-OSCR (%)**	**Unknown AUROC (%)**	**Unknown AUPR (%)**		
Resting state	96.37 ± 1.10	97.97 ± 1.10	97.23 ± 1.30		
Transient sensory stimulation	97.36 ± 0.43	98.74 ± 0.84	97.66 ± 1.13		
Steady-state sensory stimulation	96.34 ± 1.00	98.16 ± 1.47	97.22 ± 1.57		
P300 Oddball	96.67 ± 0.75	98.31 ± 1.21	97.36 ± 1.50		
Motor execution	96.58 ± 0.63	98.02 ± 1.24	96.78 ± 1.45		
**(C) Subject-level omnibus tests of paradigm effects on DIR.**					
**Metric**	**Analysis unit**	**Friedman $\chi^{2}$**	***p* value**	**Kendall’s W**	
DIR@FPIR = 5%	Subject level, N=95	105.56	$6.43\times 10^{-22}$	0.278	
DIR@FPIR = 1%	Subject level, N=95	208.01	$7.12\times 10^{-44}$	0.547	

Values in Panels A and B are mean ± standard deviation across 10 identity-split repetitions. Feature and classifier selection was performed using enrolled identities only. Shrinkage LDA was selected in all 10 repetitions; DE was selected in eight repetitions and DE + RELPSD in two repetitions.

## Data Availability

The data analyzed in this study are available from the publicly accessible M3CV database described in the original M3CV publication [16].

## Supplementary Materials

The following supporting information can be downloaded at: https://www.mdpi.com/article/10.3390/s26175471/s1, Figure S1A: Feature-set-by-paradigm comparison under the P4 protocol. Cell values indicate Rank-1 accuracy; Figure S1B: Model-by-paradigm comparison under the P4 protocol. Cell values indicate Rank-1 accuracy; Figure S2A: Robustness ranking of the five paradigms across the predefined analytical pipelines. (A) Mean rank, with smaller values indicating better performance. (B) Number of first-place rankings among the 11 pipelines; Figure S2B: Within-paradigm contributions of the individual tasks under the P4 protocol; Table S1: Within-session auxiliary verification under the predefined primary pipeline; Table S2: Closed-set statistical analysis and conventional-pipeline robustness; Table S3: Conventional/deep-learning robustness results; Table S4: Cross-session stress-test results; Table S5: Subject-level DIR distributions.

## Abbreviations

The following abbreviations are used in this manuscript: EEG, electroencephalography; PSD, power spectral density; RELPSD, relative power spectral density; DE, differential entropy; TS, time-statistical features; RF, random forest; LDA, linear discriminant analysis; ET, Extra Trees; LR, logistic regression; EER, equal error rate; AUC, area under the receiver operating characteristic curve; FAR, false acceptance rate; TAR, true acceptance rate; FPIR, false positive identification rate; DIR, detection and identification rate; TPIR, true positive identification rate; FNIR, false negative identification rate; OSCR, open-set classification rate; AUROC, area under the receiver operating characteristic curve; AUPR, area under the precision–recall curve.
